# Methylenetetrahydrofolate Reductase Gene rs1801133 and rs1801131 Polymorphisms and Essential Hypertension Risk: A Comprehensive Analysis

**DOI:** 10.1155/2022/2144443

**Published:** 2022-02-22

**Authors:** Yingchao Fan, Liting Wu, Wenfang Zhuang

**Affiliations:** Medical Laboratory, Shidong Hospital Affiliated to University of Shanghai for Science and Technology, No. 999, Shiguang Road, 200438 Yangpu District, Shanghai, China

## Abstract

**Background:**

Essential hypertension (EH) is a common and multifactorial disorder that is likely to be influenced by multiple genes. The methylenetetrahydrofolate reductase (*MTHFR*) gene rs1801133 and rs1801131 polymorphisms influence MTHFR enzyme activity and plasma homocysteine concentration. In addition, variations in MTHFR functions likely play roles in the etiology of EH. Thus far, a large number of studies investigating the associations between the *MTHFR* polymorphisms and EH have provided controversial or inconclusive results. To better assess the purported relationship, we performed a comprehensive analysis of 52 published studies. *Objective and Methods*. Eligible studies were identified by searching the PubMed, Wanfang, and China National Knowledge Infrastructure (CNKI) databases. Odds ratios (ORs) with 95% confidence intervals (CIs) were estimated to assess the potential association between the *MTHFR* rs1801133 polymorphism and EH.

**Results:**

Overall, 10712 patients and 11916 controls were involved; we observed significantly increased association between the *MTHFR* rs1801133 polymorphism and EH risk (such as T vs. C: OR = 1.38, 95% CI = 1.25 − 1.54, *P* ≤ 0.001), with similar results evident within race subgroups (such as Asian: T vs. C: OR = 1.47, 95% CI = 1.30 − 1.67, *P* ≤ 0.001; compared to Chinese: T vs. C: OR = 1.54, 95% CI = 1.33 − 1.79, *P* ≤ 0.001). Similar associations were also found in subgroups defined by the source of controls and genotype methods. To our regret, based on the limited studies, no association was detected for rs1801131 polymorphism.

**Conclusions:**

Our study provides evidence that the *MTHFR* rs1801133 null genotype may increase EH risk. Future studies with larger sample sizes are warranted to evaluate this association in more detail.

## 1. Introduction

Essential hypertension (EH) has a high prevalence rate worldwide and is considered to derive from complex interactions between diverse genes and environmental conditions [1, 2]. The present evidence-based treatment of EH is a critical intervention in reducing cardiovascular (CV) morbidity and mortality [3]. A contemporary meta-analysis of 123 studies with 613,815 hypertensive participants showed that, for every 10 mmHg reduction in systolic blood pressure, there is a significantly decreased risk of major CV disease events (relative risk 0.80 and 95% confidence interval (CI) 0.77–0.83), coronary heart disease (0.83 and 0.78–0.88), stroke (0.73 and 0.68–0.77), and heart failure (0.72 and 0.67–0.78) [4]. EH accounts for 90%-95% of all patients with hypertension, and approximately 20%-60% of its etiology is influenced by genetic factors [5].

Most people with hypertension do not present with typical symptoms, making the condition easy to ignore and reducing the opportunities for early medical intervention. Therefore, the identification of high-risk patients is particularly meaningful, since identifying such patients with high blood pressure as early as possible allows for timely monitoring of blood pressure, regular follow-ups, and options for improving unhealthy lifestyles. In known high-risk patients, rising blood pressure can be immediately detected and treatment can be provided timeously, thus avoiding complications and, ultimately, reducing the incidence of hypertension and improving quality of life [3, 6, 7].

Studies have shown that a high plasma concentration of homocysteine (Hcy) may injure the vascular endothelium, which results in hypertension and a predisposition toward atherosclerosis. Elevated Hcy levels have been identified as an independent risk factor for hypertension [8–11]. Methylenetetrahydrofolate reductase (MTHFR) plays an important role in the metabolism of Hcy. The rs1801133 polymorphism of the *MTHFR* gene is a C to T transition at nucleotide position 677 (C667T) in exon 4, which results in a change from alanine to valine at amino acid 222. This mutation may lead to a decrease in MTHFR activity and heat tolerance and thus metabolic damage of Hcy, which may moderately increase plasma Hcy levels [12, 13]; moreover, 1298 (A to C) also may cause a significant reduction in enzyme activity. This information suggests that *MTHFR* two polymorphisms may be related to EH development and susceptibility. It is therefore worthwhile to demonstrate whether there is an association between this polymorphism and EH risk, as it may provide guidance for the prevention and diagnosis of EH in the clinic. Thus far, numerous studies have reported the association between the *MTHFR* two common polymorphisms and EH risk. We therefore performed a comprehensive analysis of 52 different case-control studies to derive a convincing conclusion regarding this apparent association [14].

## 2. Materials and Methods

### 2.1. Identification of Eligible Studies

Searches were performed in the PubMed, Wanfang, and China National Knowledge Infrastructure (CNKI) databases (updated on Dec. 10, 2021) using the following related keywords: polymorphism/variant/mutation, hypertension/essential hypertension, and MTHFR/methylenetetrahydrofolate reductase. We included all studies that described a relationship between the *MTHFR* two polymorphisms and EH susceptibility. All of the included studies met the following criteria: (1) association between *MTHFR* two polymorphisms and EH risk; (2) case-control study; (3) each genotype frequency is shown in tables; and (4) genotype distributions of the control were consistent, with a Hardy-Weinberg equilibrium (HWE) more than 0.05. Otherwise, studies should be excluded with the following issues: (1) no control, (2) incomplete genotype frequency data, (3) duplication studies, and (4) not according with HWE in control groups.

### 2.2. Data Extraction

We collected the following information in our analysis: first author's last name, year of publication, ethnic origin, sample size (cases/controls), study design, HWE of controls and genotype methods, and genotype frequencies in cases/controls.

### 2.3. Quality Assessment

In this meta-analysis, the quality was assessed using the Newcastle-Ottawa Scale (NOS) for cross-sectional study quality assessment. The methodological quality of each study (sampling strategy, response rate, and representativeness of the study), comparability, and outcome were checked using the NOS tool. Studies with a score of more than 7 out of 10 were considered as good quality. This cut-off point was determined after reviewing relevant meta-analyses from the literature [15–17]. Moreover, to assess the quality of each qualified articles by quality score to explore other potential sources of heterogeneity, another score of quality assessment was applied. The quality scores of the studies ranged from 0 (lowest) to 15 (highest). Studies with scores ≤ 9 were categorized into low quality, while those with scores > 9 were considered as high quality [18].

### 2.4. Statistical Analysis

The extracted data were imported into the Stata software program (version 10.0, Corporation, College Station, Texas) for analysis. Odds ratios (ORs) with 95% CIs were used to measure the strength of the association [19, 20]. The subgroup analysis stratified by race was performed first. Race was categorized as European, Asian, Mixed, Chinese, and non-Chinese (all people who are not Chinese) subtypes. The source of the control subgroups was defined based on two classifications: hospital-based (HB) and population-based (PB). For *MTHFR* rs1801133 and rs1801131, we investigated the relationship between genetic variants and EH risk in five different models (T-allele vs. C-allele or A-allele vs. C-allele, TT vs. CC or AA vs. CC, TC vs. CC or AC vs. CC, TT+TC vs. CC or AA+AC vs. CC, and TT vs. TC+CC or AA vs. AC+CC).

The evaluation of heterogeneity within the included studies was assessed using Cochrane's *Q* test (chi-square) and *I*^2^ (%) statistical analysis. A fixed effect model was applied when the effects were assumed to be homogenous (*P* > 0.05, *I*^2^ ≤ 50%); otherwise, the random-effect model was adopted (*P* < 0.05, *I*^2^ ≥ 50%) [21, 22]. When heterogeneity was observed, the source of the heterogeneity was explored via subgroup analysis, performed using the ethnicity, publication year, study design, and genotype methods.

The presence of potential publication bias was determined using the Egger/Begg's test and presented graphically in the form of a funnel plot [23]. In addition, the departure of *MTHFR* polymorphism frequencies from expectations under HWE were assessed in controls using the Pearson chi-square test [24]. Another sensitivity analysis was conducted to assess the stability of the results. Finally, the power and sample size analysis of our meta-analysis was calculated using a program called PS: Power and Sample Size Calculation (http://biostat.mc.vanderbilt.edu/wiki/Main/PowerSampleSize#Windows) [25].

### 2.5. Genotyping Methods

Genotyping to identify single nucleotide polymorphisms (SNPs) in the *MTHFR* gene was conducted using the following analyses: polymerase chain reaction-restriction fragment length polymorphism (PCR-RFLP), TaqMan, sequencing, PCR, amplification refractory mutation system-PCR (ARMS-PCR), and high-resolution melt (HRM) genotyping.

### 2.6. Metaregression

Random-effect metaregression analysis was performed to define the source of publication bias, with the subgroups of publication year, ethnicity, source of control, and genotype method set as independent variables and the log values considered as dependent variables [26].

### 2.7. Gene Interaction Network Analysis of the *MTHFR* Gene

In order to fully understand the role of *MTHFR* and its potential functional partners in EH, we used the STRING online server (http://string-db.org/) to construct a *MTHFR* gene-gene interaction network [27, 28].

## 3. Results

### 3.1. Study Selection and Characteristics in our Meta-analysis

**Figure 1:**
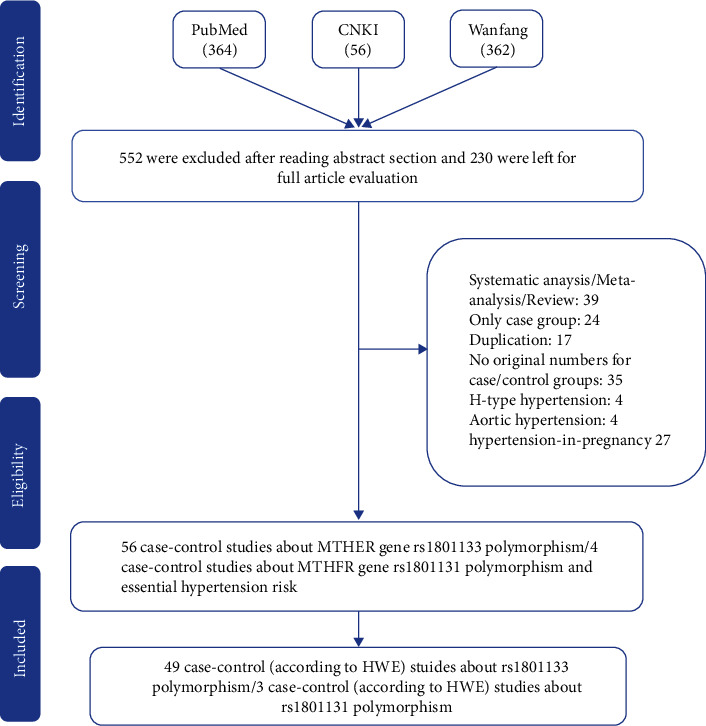
Flowchart illustrating the search strategy used to identify association studies for *MTHFR* gene rs1801133 polymorphisms and EH risk.

**Figure 2:**
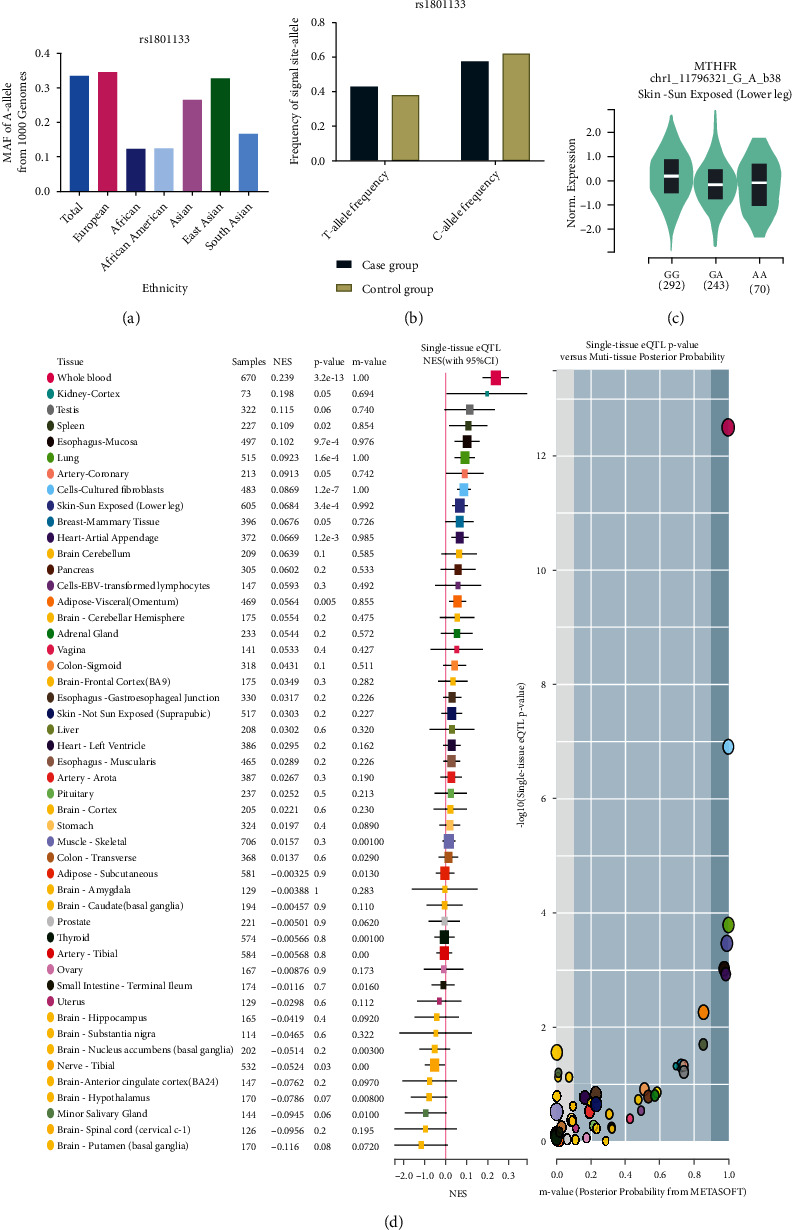
(a) The MAF of minor allele (mutant allele) for *MTHFR* gene rs1801133 polymorphism from the 1000 Genomes online database. (b) The frequency about T-allele or C-allele both in the case and control groups. (c) The distribution of each genotype from online GTEx Portal (https://www.gtexportal.org/home/). (d) The risk frequency of rs1801133 polymorphism to several diseases from TCGA database.

**Figure 3:**
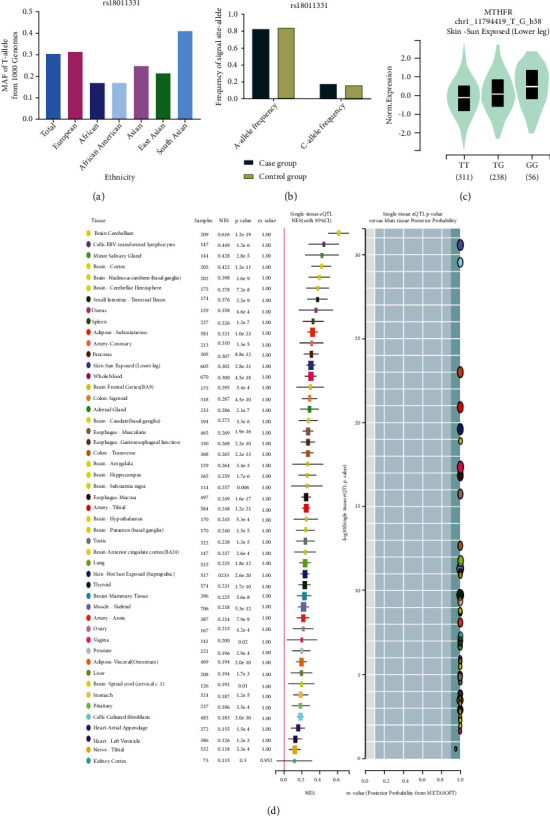
(a) The MAF of minor allele (mutant allele) for *MTHFR* gene rs1801131 polymorphism from the 1000 Genomes online database. (b) The frequency about T-allele or C-allele both in the case and control groups. (c) The distribution of each genotype from online GTEx Portal (https://www.gtexportal.org/home/). (d) The risk frequency of rs1801131 polymorphism to several diseases from TCGA database.

Using appropriate keywords (see Materials and Methods), we identified 364 articles from PubMed, 56 from CNKI, and 362 from the Wanfang database. In total, 552 articles were excluded after review of the abstract, leaving 230 articles for full article evaluation. Among them, 39 articles featured systematic analysis/meta-analysis/review; 24 covered only case groups; 17 articles were duplicates of other papers; 35 had no original numbers for case/control groups and presented only total numbers; 28 articles focused on H-type hypertension; 4 were related to aortic hypertension; and 27 covered hypertension in pregnancy (Figure 1). After exclusion of the above studies by full article review, we were left with 55 articles covering 60 case-control studies, of which 8 case-control studies were not consistent with HWE and were excluded. In total, 49 case-control studies about rs1801133 and 3 case-control studies about rs1801131 were included in our current analysis. All essential information is listed in Table 1, including first author, publishing year, race, the numbers of cases and controls, HWE, genotype numbers in cases/controls, study design, and genotype methods. Our study comprised 9 European, 36 Asian, and 4 Mixed-race case-control studies. The T frequency was 43.59% in the Asian group, 35.2% among Europeans, and 47.7% in the Mixed-race group, which indicated that the European group had lower frequency of T-allele than the Asian and Mixed groups (*P* < 0.05). The distribution of genotypes in all the controls was in agreement with HWE. In addition, we confirmed the minor allele frequency (MAF) reported for the seven main worldwide populations in the 1000 Genomes Browser [29] (https://www.ncbi.nlm.nih.gov/snp/rs1801133), as follows: Global (0.335), European (0.345), East Asian (0.328), South Asian (0.167), African (0.123), African American (0.125), and Asian (0.265) (Figure 2(a)). When we examined the frequency of T- and C-alleles both in the case and control groups, our analyses showed similar allele frequencies in each group (Figure 2(b)). Finally, we used The Cancer Genome Atlas (TCGA) database to search for trends in the frequency of rs1801133 polymorphisms. Our results indicated that the TT (AA) frequency was relatively low compared to other genotypes (Figure 2(c)). This polymorphism is associated with the coronary artery, rather than the aorta artery left ventricle and the tibial artery (https://www.gtexportal.org/home/) (Figure 2(d)). We also showed the corresponding information about rs1801131 polymorphism (Figures 3(a)–3(d)).

### 3.2. Quantitative Data Synthesis

**Figure 4:**
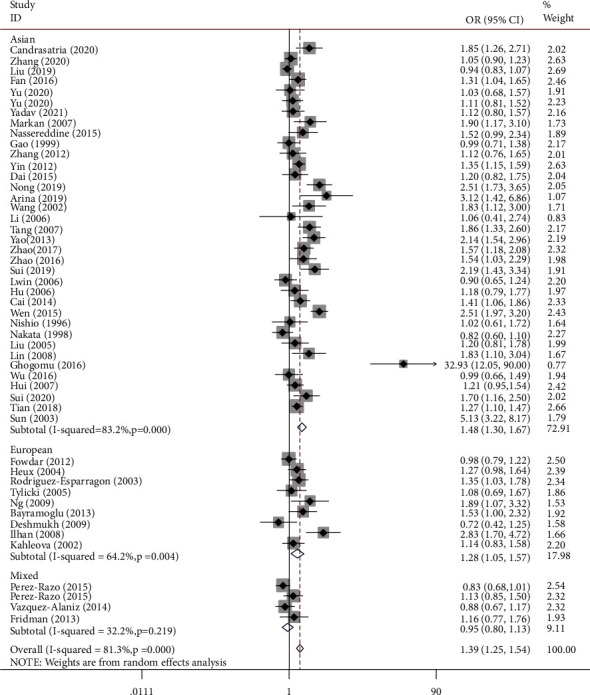
T-allele frequencies for the *MTHFR* gene rs1801133 polymorphism among cases/controls stratified by subgroups in T-allele vs. C-allele model in regular ethnic subgroup.

**Figure 5:**
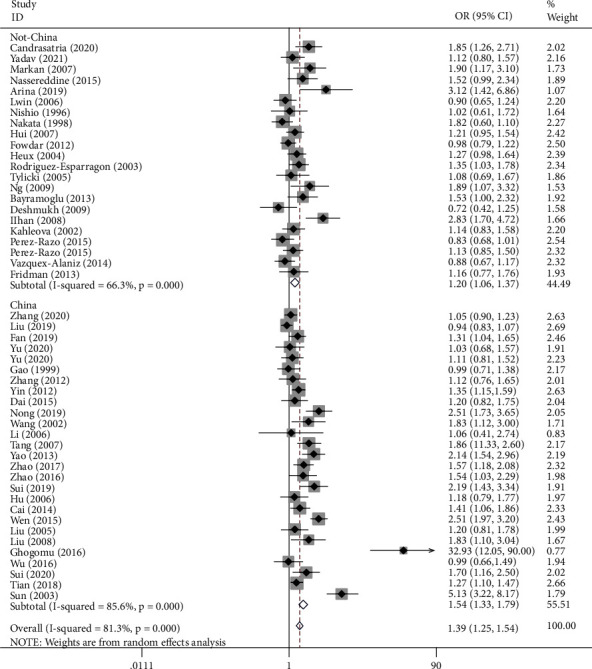
T-allele frequencies for the *MTHFR* gene rs1801133 polymorphism among cases/controls stratified by subgroups in T-allele vs. C-allele model in Chinese/non-Chinese subgroup.

**Figure 6:**
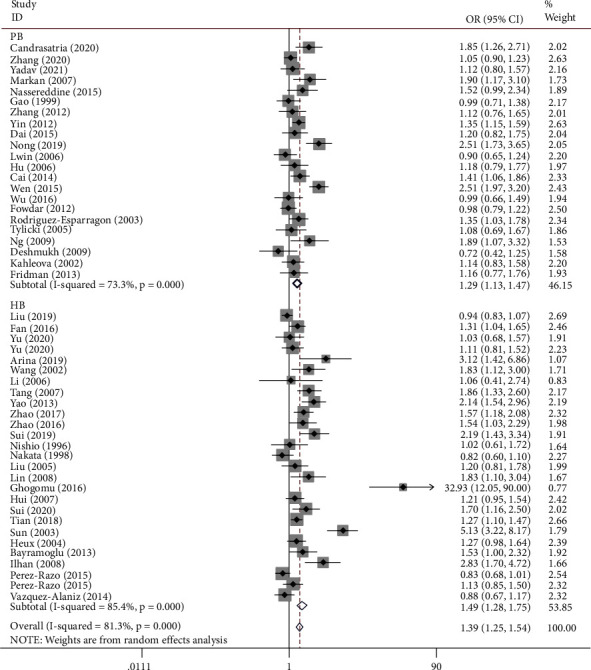
T-allele frequencies for the *MTHFR* gene rs1801133 polymorphism among cases/controls stratified by subgroups in T-allele vs. C-allele model in source of control subgroup.

**Figure 7:**
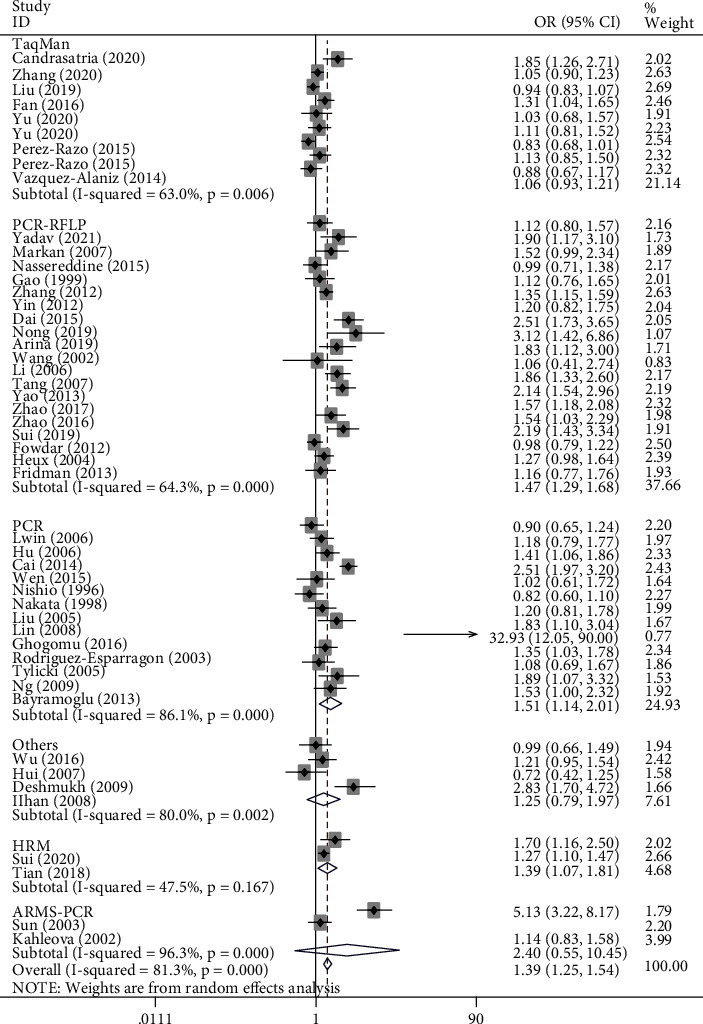
T-allele frequencies for the *MTHFR* gene rs1801133 polymorphism among cases/controls stratified by subgroups in T-allele vs. C-allele model in genotype method subgroup by random-effect model.

**Figure 8:**
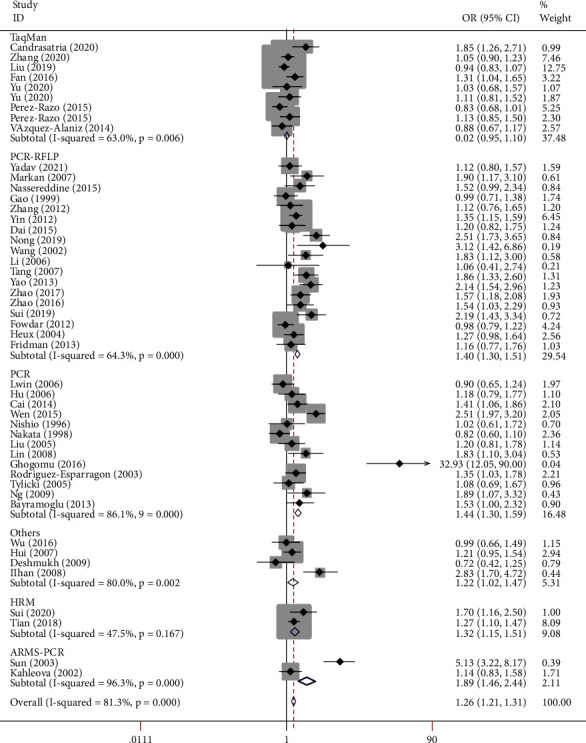
T-allele frequencies for the *MTHFR* gene rs1801133 polymorphism among cases/controls stratified by subgroups in T-allele vs. C-allele model in genotype method subgroup by fixed effect model.

Table 2 shows the summary of odds ratios of *MTHFR*, based on 10,712 EH cases and 11,916 matched controls. We observed an increased association between the *MTHFR* rs1801133 polymorphism and EH in total population groups (for example, T-allele vs. C-allele: OR = 1.38, 95% CI = 1.25 − 1.54, P_h_ < 0.001, *P* < 0.001, *I*^2^ = 81.3%, Figure 4). Similar trends were observed in the analyses of ethnic subgroups (T-allele vs. C-allele: OR = 1.47, 95% CI = 1.30 − 1.67, P_h_ < 0.001, *P* < 0.001, *I*^2^ = 83.2% for Asians, Figure 4; OR = 1.28, 95% CI = 1.05 − 1.57, P_h_ = 0.004, *P* = 0.014, *I*^2^ = 64.2% for Europeans, Figure 4; OR = 1.54, 95% CI = 1.33 − 1.79, P_h_ < 0.001, *P* < 0.001, *I*^2^ = 85.6% for Chinese, Figure 5; and OR = 1.20, 95% CI = 1.06 − 1.37, P_h_ < 0.001, *P* = 0.004, *I*^2^ = 66.3% for non-Chinese). In order to analyze the source of controls and determine the source of heterogeneity, the ratios were calculated for the HB and PB subgroups. The results showed significantly increased relationships in these groups (T-allele vs. C-allele: OR = 1.49, 95% CI = 1.28 − 1.75, P_h_ < 0.001, *P* < 0.001, *I*^2^ = 85.4% for HB and OR = 1.29, 95% CI = 1.13 − 1.47, P_h_ < 0.001, *P* < 0.001, *I*^2^ = 73.3% for PB) (Figure 6). As different methods for detecting this polymorphism were applied in all of the included studies, we considered whether positive results were associated with particular genotyping methods. Our analyses revealed some significant findings, such as PCR (T-allele vs. C-allele: OR = 1.51, 95% CI = 1.14 − 2.01, P_h_ < 0.001, *P* = 0.004, *I*^2^ = 86.1%), PCR-RFLP (T-allele vs. C-allele: OR = 1.47, 95% CI = 1.29 − 1.68, P_h_ < 0.001, *P* < 0.001, *I*^2^ = 64.3%, Figure 7), and HRM analyses (T-allele vs. C-allele: OR = 1.32, 95% CI = 1.15 − 1.51, P_h_ < 0.001, *P* < 0.001, *I*^2^ = 47.5%, Figure 8). To our regret, no positive associations were observed for rs1801131 polymorphism (Table 2).

### 3.3. Publication Bias and Sensitivity Analysis

**Figure 9:**
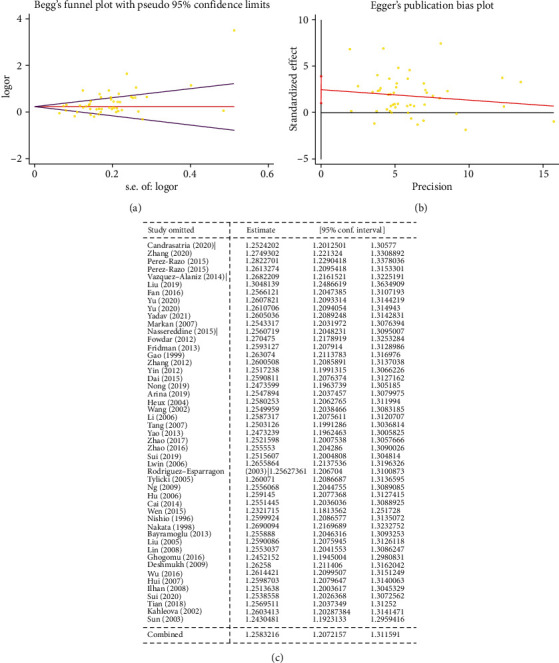
(a) Begg's funnel plot for publication bias test (T-allele vs. C-allele). (b) Egger's publication bias plot (T-allele vs. C-allele). (c) Sensitivity analysis (T-allele vs. C-allele).

Begg's funnel plot and Egger's test were performed to determine the publication bias of the included studies. Significant obvious evidence of publication bias was detected in five genetic model analyses (such as Figures 9(a) and 9(b) regarding T-allele vs. C-allele) (Table 3).

To remove studies which may influence the power and stability of the current meta-analysis, sensitivity analysis was applied, but no sensitive case-control studies were found for this SNP among the above five models (such as Figure 9(c) regarding T-allele vs. C-allele).

### 3.4. Metaregression

**Figure 10:**
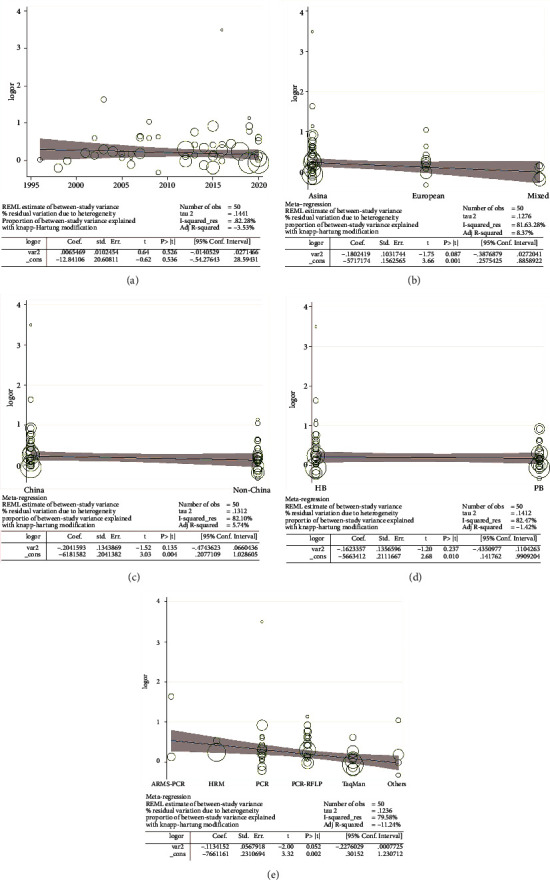
Random-effect metaregression of log odds ratio versus publication year (a), regular ethnicity (b), Chinese/non-Chinese (c), source of control (d), and genotype methods (e), respectively, in EH.

The metaregression analysis indicated that there is a significant relationship for the allele model (T-allele vs. C-allele) with respect to ethnicity, source of control, and genotype methods, with a regression coefficient of 0.001, 0.004, 0.010, and 0.002, respectively. There was no association with publication year, which suggests that the heterogeneity from the rs1801133 polymorphism in EH may be due to the ethnicity, source of control, and genotype method subgroups (Figures 10(a)–10(e)).

### 3.5. Gene-Gene Network Diagram and Interactions

**Figure 11:**
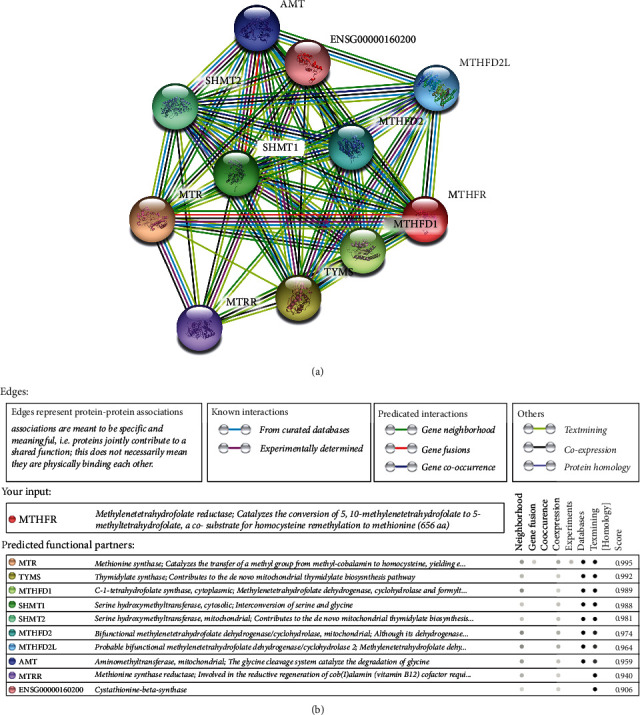
Human MTHFR interaction network with other genes obtained from STRING server. At least 10 genes have been indicated to correlate with MTHFR gene. (a) The gene-gene interaction. (b) The detail of relative ten core genes.

Our analysis using the STRING online server indicated that *MTHFR* interacts with numerous genes. The ten most significant genes from the network of gene-gene interactions have been listed in Figures 11(a) and 11(b). These ten genes are methionine (*MTR*), thymidylate synthase (*TYMS*), C-1-tetrahydrofolate synthase (*MTHFD1*), serine hydroxymethyltransferase 1 (*SHMT1*), serine hydroxymethyltransferase 2 (*SHMT2*), bifunctional methylenetetrahydrofolate dehydrogenase (*MTHFD2*), probable bifunctional methylenetetrahydrofolate dehydrogenase (*MTHFD2L*), aminomethyltransferase (*AMT*), and methionine synthase reductase (*MTRR*).

## 4. Discussion

While the precise causes of hypertension are still unknown, the risk factors include genetic factors, age, and unhealthy lifestyle practices, with 70-80% of hypertensive cases resulting from unhealthy lifestyle practices. As the risk factors for high blood pressure accumulate, the risk of high blood pressure increases [30, 31].

The detection of significant polymorphisms may be a suitable method to predict the risk of hypertension in susceptible individuals. Our current study focused on EH, a common type of hypertension, and included 10,712 patients with EH and 11,916 healthy individuals. In the overall analysis, we observed that individuals carrying TT or a T-allele may have an increased risk of developing EH compared to those with CC or a C-allele (between 37% and 89%). In other words, individuals carrying T-allele or TT genotype may have more possibility to suffer from hypertension in a current or in future time. This can give these people warnings, such as regular changes in blood pressure, changing bad habits, moderating physical exercise, or early medical intervention. Significant heterogeneity was indicated in all of the genetic models. To determine the source of the variation, we analyzed the associations in other subgroups, such as ethnicity, source of control, and genotype method. In parallel, significant relationships were also observed with the ethnicity, source of control, and genotype method subgroups, which provided further support for hypothesis that the rs1801133 polymorphism is a risk factor for EH. In addition, when we used metaregression analysis to evaluate the source of heterogeneity, the aforementioned three subgroups emerged as significant sources of variation. The power of our study was 1, suggesting that our conclusions were accurate.

Several meta-analyses regarding the rs1801133 polymorphism and hypertension have been published to date. For example, Wu et al. included 30 case-control studies, and their findings supported a role for the rs1801133 polymorphism in the risk of developing EH [32]. Kosmas et al. identified 23 comparisons relating to hypertensive disease in pregnancy, and they concluded that the T-allele of the rs1801133 polymorphism may increase the risk of hypertension in pregnancy by 1.21-fold [33]. Qian et al. combined 26 published studies of both hypertension and hypertension in pregnancy, suggesting that the rs1801133 polymorphism may be one independent risk factor [34]. Finally, Yang et al. performed a meta-analysis of 27 studies, including 5,418 EH and 4,997 controls, and their findings supported the evidence that carriers of the rs180113 T-allele were susceptible to EH [35]. However, the above studies were subject to some disadvantages. First, in several studies, the HWE values were not consistently above 0.05, which may have increased the heterogeneity and reduced the power of the conclusions. Second, each study omitted other related case-control studies, whereas our current study is a relative comprehensive analysis. Third, some articles did not distinguish the types of hypertension, which may have introduced variability because different kinds of hypertension have different etiologies, pathogenesis, and genetic backgrounds. For these reasons, we focused on one form of hypertension for our analysis. Fourth, our analysis increased genotype subgroup and evaluation of power. Fifth, we analyzed gene-gene interactions between *MTHFR* and related genes to elucidate potential functional interactions. Despite these disadvantages, it should be noted that the conclusions from our current study are similar to those from previous meta-analyses.

A key feature of our study was the identification of gene-gene interactions for *MTHFR*. The average scores of the ten most significant genes were more than 0.9. The top three genes that were suggested to interact with *MTHFR* were *MTR* (0.995), *TYMS* (0.992), and *MTHFD1* (0.989). MTHFR and MTR both participate in homocysteine metabolism, regulating different stages of the process. MTHFR converts 5,10-methylene-THF into 5-methyl-THF, while MTR catalyzes the demethylation of 5-methyl-THF to THF and the remethylation of homocysteine to methionine [36, 37]. The MTR 2756 A/G polymorphism is also associated with the risk of hypertension [38].

Some limitations in our meta-analysis should be considered. First, our analyses indicated that the heterogeneity identified in our study emerged from ethnicity, source of control, and genotype methods. Future studies should optimize the design of both retrospective and prospective research projects to overcome this deficiency. Second, EH is a complex disease including genetic and other factors (such as environment, diet, and concomitant disease) [39], and future studies should analyze the gene-gene or gene-environment interactions using larger sample sizes. Third, further meta-analyses should cover all kinds of hypertension, analyzing the associations and determining the genetic background of each type individually. Fourth, the specific mechanism underpinning the impact of the rs1801133 or rs1801131 polymorphism should be explored, for example, using animal models of EH.

## 5. Conclusion

Our meta-analysis provides evidence that the *MTHFR* 677T null genotype is associated with increased risk of EH, suggesting that further, well-designed large studies are necessary to confirm this relationship. Furthermore, it will be important to focus on the mechanism of action of the rs1801133T-allele to explain the complete chain of evidence for the prevention of EH in the future.

## Figures and Tables

**Table 1:** Characteristics of studies of *MTHFR* gene two common polymorphisms and essential hypertension risk included in our meta-analysis.

Author	Year	Country	Ethnicity	Case	Control	Case			Control			SOC	HWE	Genotype	Score	NOS
						TT	TC	CC	TT	TC	CC					
Gao	1999	China	Asian	127	170	15	68	44	24	84	62	PB	0.6	PCR-RFLP	7	7
Wang	2002	China	Asian	105	46	37	51	17	9	23	14	HB	0.935	PCR-RFLP	7	6
Sun	2003	China	Asian	55	173	27	22	6	18	69	86	HB	0.456	ARMS-PCR	6	6
Liu	2005	China	Asian	100	100	26	45	29	19	50	31	HB	0.883	PCR	7	7
Li	2006	China	Asian	26	30	2	6	18	2	7	21	HB	0.226	PCR-RFLP	7	6
Hu	2006	China	Asian	110	115	16	39	55	12	42	61	PB	0.249	PCR	10	7
Tang	2007	China	Asian	252	195	20	93	139	6	51	138	HB	0.629	PCR-RFLP	8	6
Lin	2008	China	Asian	50	123	4	27	19	6	44	73	HB	0.847	PCR	7	6
Zhang	2012	China	Asian	189	165	8	53	128	7	41	117	PB	0.175	PCR-RFLP	10	8
Yin	2012	China	Asian	670	682	68	358	244	51	309	322	PB	0.096	PCR-RFLP	12	8
Yao	2013	China	Asian	150	150	49	69	32	22	67	61	HB	0.607	PCR-RFLP	8	7
Cai	2014	China	Asian	200	200	62	99	39	50	89	61	PB	0.129	PCR	10	7
Liu	2019	China	Asian	934	1075	295	439	200	356	505	214	HB	0.151	TaqMan	10	7
Ghogomu	2016	China	Asian	41	50	14	24	3	0	5	45	HB	0.709	PCR	7	6
Dai	2015	China	Asian	114	104	32	57	25	26	49	29	PB	0.562	PCR-RFLP	10	8
Wen	2015	China	Asian	174	634	76	53	45	85	291	258	PB	0.837	PCR	10	7
Wu	2016	China	Asian	123	120	11	39	73	10	40	70	PB	0.223	Gene Chip	9	7
Fan	2016	China	Asian	214	494	75	102	37	141	234	119	HB	0.493	TaqMan	9	6
Zhao	2017	China	Asian	200	200	47	99	54	29	91	80	HB	0.705	PCR-RFLP	8	7
Sui	2020	China	Asian	102	109	31	52	19	22	49	38	HB	0.397	HRM	7	6
Yu	2020	China	Asian	137	128	5	47	85	5	42	81	HB	0.877	TaqMan	8	6
Yu	2020	China	Asian	163	160	31	79	53	27	76	57	HB	0.845	TaqMan	8	7
Nong	2019	China	Asian	122	110	49	58	15	16	59	35	PB	0.267	PCR-RFLP	9	8
Zhao	2016	China	Asian	100	100	23	50	27	15	45	40	HB	0.689	PCR-RFLP	6	7
Tian	2018	China	Asian	743	718	203	373	167	148	370	200	HB	0.333	HRM	9	7
Sui	2019	China	Asian	113	73	44	50	19	10	41	22	HB	0.186	PCR-RFLP	7	7
Zhang	2020	China	Asian	741	538	164	313	264	92	268	178	PB	0.603	TaqMan	12	8
Nishio	1996	Japan	Asian	47	82	5	26	16	9	44	29	HB	0.201	PCR	5	7
Nakata	1998	Japan	Asian	173	184	19	91	63	36	83	65	HB	0.309	PCR	8	7
Lwin	2006	Japan	Asian	116	219	19	58	39	38	117	64	PB	0.215	PCR	6	8
Hui	2007	Japan	Asian	261	271	49	129	83	44	123	104	HB	0.454	PCR-SSCP	6	6
Markan	2007	India	Asian	153	133	8	40	105	0	28	105	PB	0.174	PCR-RFLP	11	8
Nassereddine	2015	Morocco	Asian	101	102	14	40	47	3	45	54	PB	0.074	PCR-RFLP	10	7
Candrasatria	2020	Indonesia	Asian	213	202	6	73	134	3	42	157	PB	0.92	TaqMan	9	7
Arina	2019	Indonesia	Asian	53	53	5	16	32	0	10	43	HB	0.448	PCR-RFLP	6	7
Kahleova	2002	Czech	European	164	173	27	55	82	18	69	86	PB	0.457	ARMS-PCR	8	8
Rodriguez-Esparragon	2003	Spain	European	232	215	34	115	83	20	100	95	PB	0.386	PCR	11	8
Heux	2004	New Zealand	European	247	249	35	125	87	25	119	105	HB	0.298	PCR-RFLP	7	7
Tylicki	2005	Austria/Poland	European	90	90	11	39	40	10	38	42	PB	0.752	PCR	11	7
Ilhan	2008	Turkey	European	78	100	10	32	36	2	26	72	HB	0.844	Real-time PCR	4	7
Deshmukh	2009	USA	European	42	118	4	16	22	18	48	52	PB	0.221	Sequencing	10	8
Ng	2009	Australia	European	38	80	10	14	14	8	32	40	PB	0.67	PCR	7	8
Fowdar	2012	Australia	European	377	393	33	174	170	35	183	175	PB	0.186	PCR-RFLP	11	7
Bayramoglu	2013	Turkey	European	125	99	22	38	65	5	38	56	HB	0.654	PCR	8	7
Fridman	2013	Argentina	Mixed	75	150	6	40	29	15	64	71	PB	0.917	PCR-RFLP	9	8
Perez-Razo	2015	Mexico	Mixed	373	391	87	174	112	101	200	90	HB	0.637	TaqMan	9	6
Perez-Razo	2015	Mexico	Mixed	199	199	67	98	34	56	108	35	HB	0.168	TaqMan	8	7
Vazquez-Alaniz	2014	Mexico	Mixed	194	194	39	93	62	43	97	54	HB	0.964	TaqMan	8	7
Yadav	2021	India	Asian	207	254	10	58	139	11	65	178	PB	0.116	PCR-RFLP	11	9

HB: hospital-based; PB: population-based; SOC; source of control; PCR-RFLP: polymerase chain reaction followed by restriction fragment length polymorphism; HRM: high-resolution melt; ARMS-PCR: amplification refractory mutation system-PCR; HWE: Hardy-Weinberg equilibrium of control group.

**Table 2:** Stratified analyses of *MTHFR* gene rs1801133 polymorphism and essential hypertension risk.

Variables	*N*	Case/control	T vs. C	TC vs. CC	TT vs. CC	TT+TC vs. CC	TT vs. TC+CC
			OR (95% CI) Ph *PI*^2^	OR (95% CI) Ph *P*	OR (95% CI) Ph *P*		
rs1801133							
Total	49	9613/10713	1.38 (1.25-1.54) 0.000 0.000 81.3%	1.26 (1.13-1.41) 0.000 0.000 58.7%	1.80 (1.47-2.20) 0.000 0.000 75.6%	1.41 (1.25-1.59) 0.000 0.000 71.1%	1.57 (1.33-1.85) 0.000 0.000 72.8%
Ethnicity							
Asian	36	7379/8262	1.47 (1.30-1.67) 0.000 0.000 83.2%	1.36 (1.19-1.55) 0.000 0.000 61.0%	1.99 (1.56-2.55) 0.000 0.000 77.6%	1.53 (1.32-1.78) 0.000 0.000 72.9%	1.67 (1.36-2.05) 0.000 0.000 76.2%
European	9	1393/1517	1.28 (1.05-1.57) 0.004 0.014 64.2%	1.11 (0.94-1.29) 0.2225 0.210 24.6%	1.73 (1.15-2.59) 0.024 0.009 54.7%	1.24 (0.99-1.54) 0.057 0.054 47.2%	1.63 (1.13-2.35) 0.045 0.009 49.4%
Mixed	4	841/934	0.93 (0.82-1.06) 0.219 0.313 32.2%	0.86 (0.69-1.08) 0.156 0.205 42.5%	1.50 (0.88-2.56) 0.042 0.133 63.4%	1.37 (0.90-2.09) 0.017 0.139 70.6%	1.06 (0.92-1.24) 0.118 0.397 48.9%
Chinese	27	6055/6762	1.54 (1.33-1.79) 0.000 0.000 85.6	1.41 (1.19-1.66) 0.000 0.000 67.4%	2.17 (1.65-2.85) 0.000 0.000 80.1%	1.63 (1.36-1.95) 0.000 0.000 77.2%	1.77 (1.41-2.25) 0.000 0.000 78.7%
Non-Chinese	22	3558/3951	1.20 (1.06-1.37) 0.000 0.004 66.3%	1.12 (0.98-1.28) 0.048 0.091 36.0%	1.35 (1.03-1.77) 0.000 0.030 58.9%	1.19 (1.03-1.38) 0.002 0.018 52.6%	1.28 (1.02-1.61) 0.002 0.035 53.3%
SOC							
HB	27	5235/5746	1.49 (1.28-1.75) 0.000 0.000 85.4%	1.38 (1.16-1.64) 0.000 0.000 67.1%	1.96 (1.47-2.67) 0.000 0.000 79.5%	1.57 (1.28-1.91) 0.000 0.000 78.1%	1.60 (1.29-1.99) 0.000 0.000 72.5%
PB	22	4378/4967	1.29 (1.13-1.47) 0.000 0.000 73.3%	1.17 (1.03-1.33) 0.019 0.019 42.7%	1.64 (1.24-2.17) 0.000 0.001 69.0%	1.28 (1.12-1.48) 0.001 0.000 55.7%	1.51 (1.16-1.97) 0.000 0.002 70.8%
Genotype methods							
PCR	13	1496/2191	1.51 (1.14-2.01) 0.000 0.004 86.1%	1.31 (0.97-1.75) 0.000 0.075 69.4%	1.93 (1.18-3.17) 0.000 0.009 78.9%	1.51 (1.10-2.07) 0.000 0.010 76.6%	1.71 (1.07-2.73) 0.000 0.024 81.1%
PCR-RFLP	19	3381/3359	1.47 (1.29-1.68) 0.000 0.000 64.3%	1.38 (1.24-1.54) 0.547 0.000 0.0%	2.14 (1.58-2.91) 0.000 0.000 60.3%	1.54 (1.33-1.78) 0.027 0.000 42.3%	1.74 (1.34-2.25) 0.003 0.000 54.2%
ARMS-PCR	2	219/346	2.39 (0.55-10.45) 0.000 0.244 96.3%	1.85 (0.87-2.47) 0.002 0.468 89.9%	5.64 (0.43-73.32) 0.000 0.186 94.3%	2.72 (0.34-21.96) 0.000 0.348 94.3%	3.72 (0.78-17.71) 0.001 0.099 90.5%
TaqMan	9	3168/3381	1.06 (0.93-1.21) 0.006 0.358 63.0%	1.01 (0.82-1.24) 0.008 0.941 61.1%	1.05 (0.85-1.30) 0.094 0.639 41.0%	1.04 (0.85-1.27) 0.005 0.691 63.9%	1.07 (0.95-1.20) 0.230 0.247 24.0%
HRM	2	845/827	1.32 (1.15-1.51) 0.167 0.000 47.5%	1.29 (1.02-1.64) 0.125 0.031 57.6%	1.76 (1.34-2.32) 0.202 0.000 38.5%	1.43 (1.15-1.78) 0.104 0.002 62.2%	1.48 (1.18-1.86) 0.609 0.001 0.0%
Other	4	504/609	1.25 (0.79-1.97) 0.002 0.346 80.0%	1.25 (0.82-1.91) 0.082 0.291 55.2%	1.45 (0.63-3.32) 0.030 0.386 66.6%	1.29 (0.78-2.15) 0.021 0.319 72.8%	1.29 (0.65-2.56) 0.081 0.468 55.5%
			A vs. C	AC vs. CC	AA vs. CC	AA+AC vs. CC	AA vs. AC+CC
			OR (95% CI) Ph *P* I-squared	OR (95% CI) Ph *P*	OR (95% CI) Ph *P*		
rs1801131	3	1099/1203	0.93 (0.80-1.09) 0.710 0.384 0.0%	0.97 (0.59-1.59) 0.990 0.901 0.0%	0.90 (0.55-1.46) 0.912 0.667 0.0%	0.92 (0.57-1.48) 0.979 0.730 0.0%	0.92 (0.77-1.11) 0.632 0.382 0.0%

P_h_: value of *Q* test for heterogeneity test; *P*: *Z* test for the statistical significance of the OR.

**Table 3:** Publication bias tests (Begg's funnel plot and Egger's test for publication bias test) for *MTHFR* gene rs1801133 polymorphism and essential hypertension risk.

Egger's test						Begg's test	
Genetic type	Coefficient	Standard error	*t*	*P* value	95% CI of intercept	*z*	*P* value
T vs. C	2.468	0.719	3.43	0.001	(1.021-3.915)	2.37	0.018
TC vs. CC	1.726	0.489	3.53	0.001	(0.743-2.709)	2.94	0.003
TT vs. CC	0.831	0.289	2.87	0.006	(0.248-1.413)	2.08	0.038
TT+TC vs. CC	1.803	0.519	3.47	0.001	(0.757-2.849)	2.96	0.003
TT vs. TC+CC	0.858	0.326	2.63	0.012	(0.201-1.514)	1.59	0.111

## Data Availability

The data used to support the findings of this study are available from the corresponding author upon request.
